# Inhalation of hydrogen gas protects against mitomycin-induced pulmonary veno-occlusive disease

**DOI:** 10.1186/s12931-024-02906-y

**Published:** 2024-07-16

**Authors:** Chenting Zhang, Yue Xing, Xuefen Wu, Qian Jiang, Xiaoyun Luo, Wei He, Shiyun Liu, Wenju Lu, Jian Wang

**Affiliations:** 1 State Key Laboratory of Respiratory Disease, National Clinical Research Center for Respiratory Disease, Guangdong Key Laboratory of Vascular Disease, Guangzhou Institute of Respiratory Health, The First Affiliated Hospital of Guangzhou Medical University, Guangzhou, Guangdong 510120 China; 2 Guangzhou Laboratory, Guangzhou International Bio Island, Guangzhou, Guangdong China; 3 Section of Physiology, Division of Pulmonary, Critical Care and Sleep Medicine, Department of Medicine, University of California San Diego, La Jolla, San Diego, CA USA

**Keywords:** Hydrogen gas, Pulmonary veno-occlusive disease, Antioxidant, Anti-inflammatory, Endothelial-to-mesenchymal transition

## Abstract

**Background:**

As a subtype of pulmonary hypertension (PH), pulmonary veno-occlusive disease (PVOD) is devastating and life-threatening disease without effective therapy. Hydrogen has been reported to exhibits antioxidant and anti-inflammatory effects in a rat model induced by monocrotaline of PH. In this study, we investigated the effects of inhaled hydrogen gas on the prevention and treatment of PVOD induced by mitomycin C (MMC) in rats.

**Methods:**

PVOD was induced in female Sprague-Dawley rats through intraperitoneal injection of MMC at a concentration of 3 mg·kg^− 1^·wk^− 1^ for 2 weeks. Inhalation of hydrogen gas (H_2_) was administered through a designed rat cage concurrently or two weeks after MMC administration. The severity of PVOD was assessed by using hemodynamic measurements and histological analysis. The expression levels of general control nonderepressible 2 (GCN2), nuclear factor erythroid 2-related factor-2 (Nrf2), heme oxygenase-1 (HO-1) and endothelial-to-mesenchymal transition (EndoMT) related proteins in lung tissue were measured. Levels of lipid peroxidation pro-inflammatory cytokines in serum were determined.

**Results:**

Inhaled H_2_ improved hemodynamics and right heart function, reversed right ventricular hypertrophy, and prevented pulmonary vessel reconstitution in both prevention and treatment approaches. It decreased malondialdehyde (MDA) levels in the serum and the expression of NADPH oxidase 1 (NOX-1) in lung tissue. It regulated Nrf2/HO-1 signaling pathway and anti-inflammatory factor GCN2 in lung tissue, accompanied by a decrease in macrophages and pro-inflammatory cytokines. Our data suggested that H_2_ inhalation effectively countered EndoMT induced by MMC, as evidenced by the detection of endothelial markers (e.g., VE-cadherin and CD31) and mesenchymal markers (e.g., vimentin and fibronectin). Further research revealed that H_2_ preserved p-Smad3 and induced p-Smad1/5/9.

**Conclusion:**

Inhalation of H_2_ effectively inhibits the pathogenesis of PVOD induced by MMC in rats. This inhibitory effect may be attributed to the antioxidant and anti-inflammatory properties of H_2_.

**Supplementary Information:**

The online version contains supplementary material available at 10.1186/s12931-024-02906-y.

## Introduction

Pulmonary veno-occlusive disease (PVOD) is a rare and devastating disease characterized by the narrowing of pulmonary venules, resulting in progressive elevation of pulmonary arterial pressure and resistance. It shares similar clinical and hemodynamic characteristics with idiopathic pulmonary arterial hypertension, which is an important subgroup of Group 1 PH. However, diagnosing PVOD is challenging due to its slow onset, often leading to delayed diagnosis and poor prognosis [1, 2]. Despite being observed and described 70 years ago, the exact mechanisms underlying PVOD remain largely unknown. Recently, the use of chemotherapeutic agents has emerged as a potential risk factor for inducing PVOD [3]. Retrospective studies have shown a strong association between exposure to chemotherapy drugs such as bleomycin, cisplatin, and mitomycin C (MMC) and increased incidence of PVOD [4, 5]. Unfortunately, there are currently no viable alternatives to these agents without side effects. Moreover, the causal role of chemotherapy in PVOD has been corroborated through the successful establishment of an MMC-induced PVOD rat model, exhibiting significant pulmonary vein occlusion and typical pathophysiological features of pulmonary hypertension, including elevated right ventricular systolic pressure (RVSP), increased Fulton index (ratio of right ventricle [RV] to left ventricle [LV] + septum [S]), and remodeling of distal pulmonary vasculature [5]. While PVOD and PH share many clinical symptoms and hemodynamic features, individuals with PVOD are considered less responsive to pulmonary vasodilator therapy due to the complications of pulmonary edema. Therefore, discovering novel and effective therapeutic strategies for PVOD is of utmost importance.

Molecular hydrogen (H_2_) is a diatomic gas that is colorless, tasteless, odorless, non-irritating, and highly flammable. It has been found to have positive effects on multiple organ systems, including the brain, heart, lung, kidney, liver, and pancreas [6–8]. H_2_ has shown protective effects against oxidative stress by mitigating inflammatory responses in diseases such as Alzheimer’s disease, hematological disorders and chronic obstructive pulmonary disease (COPD) [9, 10]. Studies have reported that inhaling H_2_ can dose-dependently alleviate lung inflammation induced by cigarette smoke in rats [10]. In addition, high-concentration H_2_ inhalation has been found to reverse small airway remodeling induced by cigarette smoke and inhibit the decline of lung function in mice [11]. Hydrogen water has also demonstrated therapeutic effects in a monocrotaline-induced PH rat model by reducing the expression of inflammatory cytokines [12].

Given the positive effects of H_2_ on various organ systems and its ability to attenuate inflammatory responses and reduce oxidative stress, H_2_ inhalation may represent a novel approach for the prevention and treatment of PH and for mitigating the side effects of chemotherapy drugs. In this study, our aim was to investigate the role of H_2_ in a rat model of MMC-induced PVOD.

## Methods

### Animal model

**Fig. 1:**
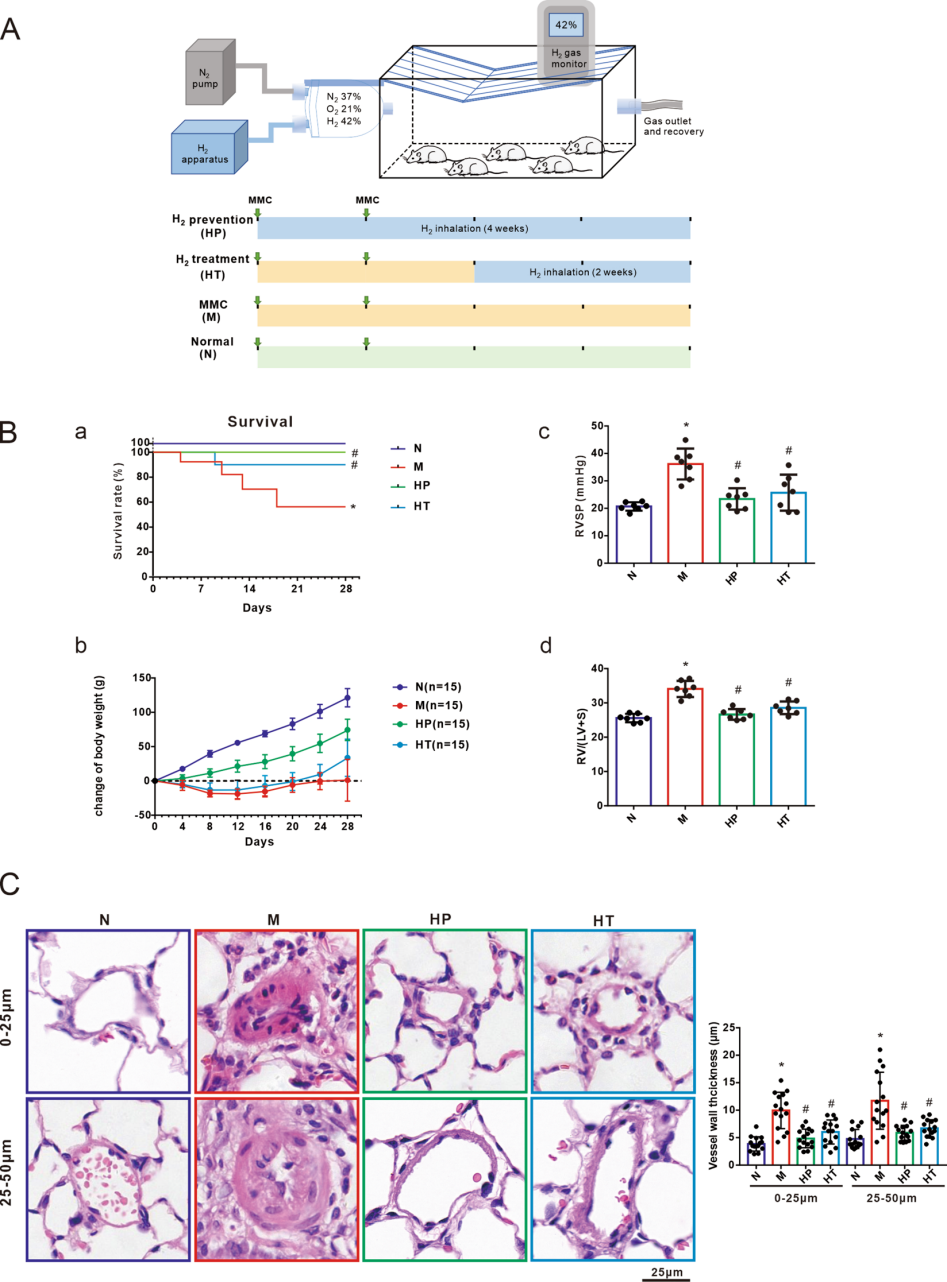
Hydrogen gas alleviates MMC-induced pulmonary veno-occlusive disease (PVOD). The rat model of PVOD was established by twice intraperitoneal injection of mitomycin C (MMC, 3 mg/kg/week). Hydrogen gas inhalation was given to the rats in a standardized mixture (42% H_2_, 21% O_2_, 33% N_2_) 1 h inhalation twice daily from the day of MMC injection and lasting for 28 days, or 14 days after MMC-injection and lasting for 14 days. **A**: Groups and procedure of different experiment schemes: Normal (N), MMC (M), H_2_ prevention (HP), H_2_ treatment (HT). The normal group of rats was exposed to air. **B**-**a**: Survival rate (*n* = 15 per group); **B**-**b**: Weight change (*n* = 15 per group); **B**-**c**: Right ventricular systolic pressure (RVSP, mmHg, *n* = 7 per group); **B**-**d**: Right ventricular hypertrophy characterized by the Fulton Index (FI, weight ratio of right ventricle [RV]/left ventricle [LV] + septum[S], *n* = 7 per group). **C**: Representative images of hematoxylin and eosin (H&E) staining and calculated Pulmonary micro-vessels thickness within the range between 0–25 μm and 25–50 μm. Scale bar represented 25 μm (zoom out) as indicated (*n* = 5 per group, 3 microscopic fields per rat). All data were presented as means ± SEM. One-way ANOVA was used for comparison among four groups. *P < 0.05 vs. Normal group, ^#^*P* < 0.05 vs. MMC group

Female Sprague-Dawley rats (150–180 g) were purchased from Guangdong Medical Experimental Animal Center (Guangzhou, China) and housed in a specific pathogen free (SPF) facility. All experimental protocols were approved by the Animal Care and Use Committee of Guangzhou Medical University and performed according to previously published procedures [5]. To establish a rat model of PVOD, the rats received intraperitoneal injections of MMC (MedChemExpress, Cat. #HY-13,316) at a dosage of 3 mg·kg^− 1^·wk^− 1^ over a period of 2 weeks. Subsequently, the rats were randomly divided into four groups: normal control group (N, *n* = 15), MMC-treated group (MMC, *n* = 15), H_2_ prevention group (HP, *n* = 15), and H_2_ treatment group (HT, *n* = 15). The rats in control group were administrated the same volume of 0.9% saline as MMC injection. In the HP group, H_2_ was administered simultaneously with the MMC injection. Conversely, in the HT group, H_2_ inhalation commenced 2 weeks after the initial MMC injection. All rats were euthanized 4 weeks after the first MMC injection. As seen in Fig. 1A, for HP group, H_2_ was administered at the same time as the MMC injection, while for HT group, H_2_ inhalation was given 2 weeks after the first injection of MMC. All number of rats (*n* = 15 per group) were used to calculated the survival rate, and the rest surviving rats were sacrificed for subsequent experiments.

### Administration of inhaled H_2_ and O_2_ to rats

Molecular H_2_ and O_2_ gases were was generated through the electrolysis of distilled water (dH_2_O) by the machine (Shanghai Asclepius Meditec Co., Ltd., Shanghai China), resulting in a fixed ratio of 66.7% H_2_ to 33.3% O_2_ (2:1 ratio). To ensure that the oxygen concentration in the rat cages remained consistent with room air and to avoid any potential effects of hypoxia or hyperoxia, the nitrogen gas (N_2_) was mixed to create an air mixture in the rodent cages containing H_2_ (42%), O_2_ (21%), and N_2_ (37%). This mixture air was delivered to the MMC-injected rats via a sealed chamber that connected to the outside air through a hole, with a low flow rate of 3.8 L/min. The rats in the normal controls (N) and MMC groups were given regular air. The concentrations of H_2_, O_2_, and CO_2_ were monitored at the beginning and end of each inhalation period to ensure the stability of each component in the air.

### Echocardiography and hemodynamic measurements

Echocardiography was performed for each group of rats (*n* = 6 in each group) using a Vevo 2100 Imaging System equipped with MS250 (13-24 MHz) linear array transducer (FUJIFILM Visual Sonics, Toronto, Canada). Right ventricular systolic pressure (RVSP) and right ventricular hypertrophy index (RVHI) were measured using the same method as we described previously (*n* = 7 in each group) [13]. After echocardiography and hemodynamic measurements, the rats were sacrificed after the removal of lung and heart under anesthesia with 3% pentobarbital sodium (30 mg/kg, i.p.).

### Enzyme-linked immunosorbent assay (ELISA)

About 4 ml of blood was extracted from the right heart by a syringe and mixed in EDTA-anticoagulant tubes followed by a low-speed centrifuge (800 g, 10 min). The plasma was obtained from the supernatant and prepared for Elisa. The concentration of IL-1β, IL-6, IL-8, IL17, TNF-α and MDA was measured by using the Elisa kit following the manufacturer’s protocol (Andygene Beijing, China).

### Histology and immunofluorescence staining

Lung slides were prepared by sectioning lung tissue samples from each group of rats for H&E staining, Masson staining and immunofluorescence staining. The fixed lungs were paraffin embedded and sectioned at 5 μm thickness. The lung slides were stained with haematoxylin and eosin (H&E staining), Masson Fontana (Masson’s staining). For immunofluorescence staining, the lung slides were dewaxed and dehydrated, followed by antigen retrieval in boiling EDTA antigen retrieval buffer. Then the lung slides were incubated with macrophagocyte marker CD68 (Cell Signaling, Cat. #97778), smooth muscle cell marker α-SMA (Sigma-Aldrich, Cat. #A5228) and the endothelial cell marker vWF (Sigma-Aldrich, Cat. #F3520). Two or three microscopic fields from each lung slide of different groups (*n* = 5 per group) were randomly chosen for immune cell counting and fluorescence intensity measurement.

### Western blotting

Lung tissue samples were harvested and mixed with radioimmunoprecipitation assay (RIPA) lysis buffer containing phenyl methane sulfonyl fluoride (PMSF) and phosphatase inhibitors to isolate proteins. The samples were then centrifuged at 10,000 g for 15 min, and the supernatant was collected for protein quantitation using a bicinchoninic acid (BCA) assay. Equal amount of total protein from each group was loaded and separated on SDS-PAGE (Bio-Rad), transferred onto polyvinylidene difluoride membranes and the membranes were then incubated with anti-p-Smad3 (Cell Signaling, Cat. #9520), anti-p-Smad1/5/9 (Cell Signaling, Cat. #13820) anti-Vimentin (Cell Signaling, Cat. #5741), anti-FN1 (Proteintech, Cat. #15613-1-AP), anti-CD31 (Proteintech, Cat. #11265-1-AP), anti-VE-cadherin (Invitrogen, Cat. #36-1900), anti-GCN2 (Cell Signaling, Cat. #3302), anti-β-actin (Santa Cruz Biotechnology, Cat. #sc-47,778), HO-1 (Abcam, Cat. #ab305290), Nrf-2 (Abcam, Cat. #ab313825), NOX-1 (Abcam, Cat. #ab131088) overnight at 4 °C. Membranes were washed by TBST and incubated in anti-rabbit or anti-mouse secondary antibodies for 1 h at room temperature. Band intensity was quantified by using ImageJ and represented as arbitrary units. The relative protein levels were normalized to the housekeeping protein β-actin.

## Results

### Inhalation of H_2_ significantly improved the histological and hemodynamic changes in MMC-induced PVOD rat model

The administration of H_2_ through inhalation improved the hemodynamic changes induced by MMC and significantly enhanced survival. The MMC treatment led to increased mortality rate and decreased body weight. However, inhalation of H_2_ effectively reversed the high mortality and weight loss caused by MMC in both prevention and treatment group (Fig. 1B-a, b).

Consistent with previous findings [5, 13], the exposure to MMC resulted in a significant increase in right ventricular systolic pressure (RVSP) and Fulton index (ratio of right ventricle [RV] to left ventricle [LV] + septum [S]). However, the application of H_2_, in both the prevention and treatment groups, reversed the hemodynamic changes induced by MMC, indicating therapeutic effects (Fig. 1B-c, d).

Furthermore, histological analyses revealed significant pulmonary microvascular remodeling following MMC stimulation, characterized by an increase in the medial area of vasculature. However, inhalation of H_2_ in both the prevention and treatment groups mitigated MMC-induced pulmonary vascular remodeling. This suggests that H_2_ inhalation has a protective effect against MMC-induced changes in pulmonary vasculature (Fig. 1C).

### Inhalation of H_2_ significantly improved the right heart function in MMC-induced PVOD rat model

**Fig. 2:**
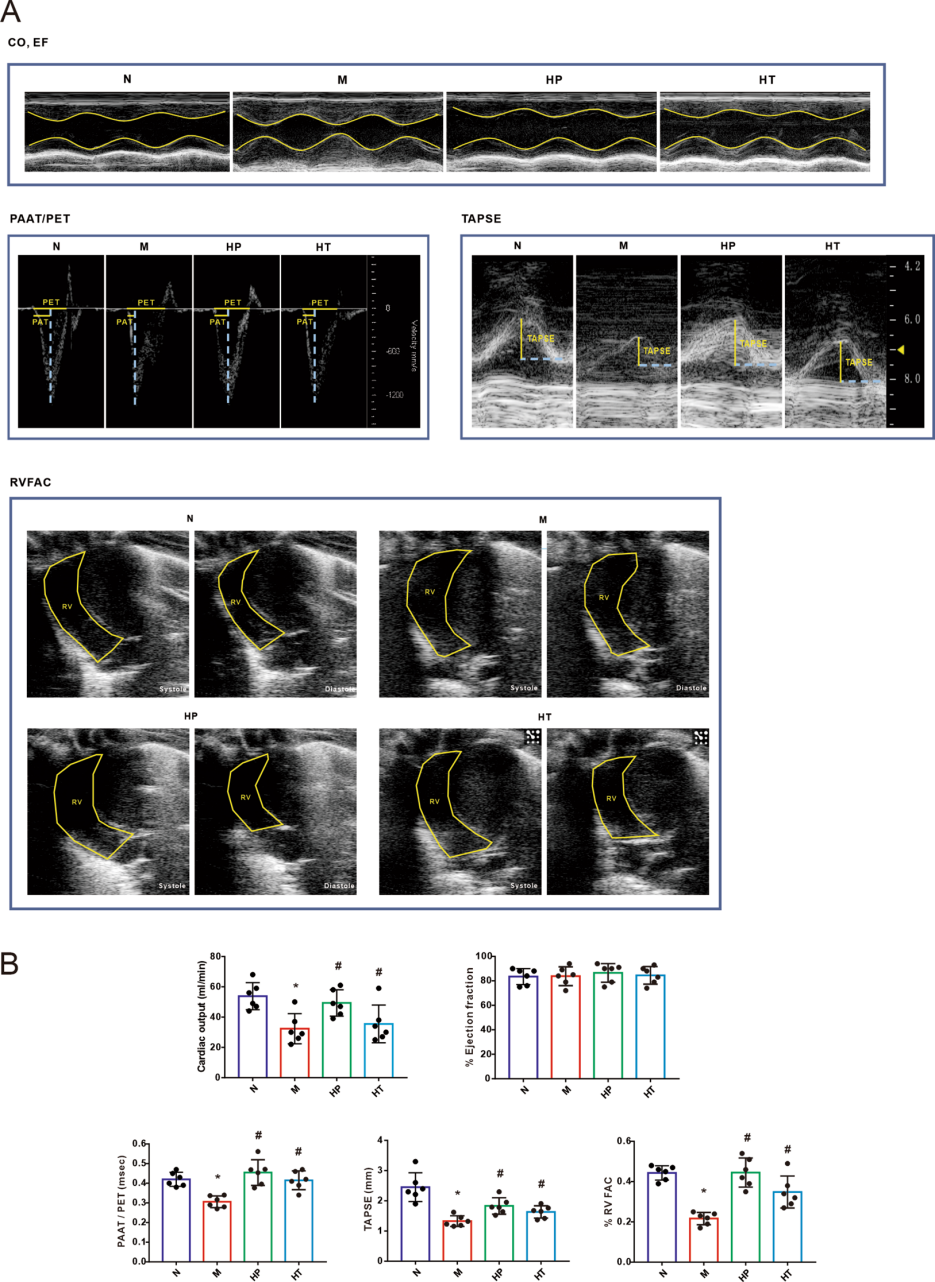
Hydrogen gas protects the right ventricular function in MMC-induced PVOD rats. **A**-**B**: Representative images and analyzed data of the rat echocardiography., CO: Cardiac output; EF: ejection fraction; PAAT/PET: pulmonary acceleration time/pulmonary ejection time. TASPE: Tricuspid annular plane systolic excursion. RV FAC: RV fractional area change. Normal (N), MMC (M), H_2_ prevention (HP), H_2_ treatment (HT). Date was represented as means ± SEM, *n* = 6 per group. One-way ANOVA was used for comparison among four groups. ^*^P < 0.05 vs. Normal, ^#^*P* < 0.05 vs. MMC

Echocardiographic analysis was conducted to assess the impact of H_2_ inhalation on right heart function in a rat model of MMC-induced PVOD. The results demonstrated that MMC administration led to substantial impairment in right heart function, as evidenced by reduced cardiac output (CO), tricuspid annular plane systolic excursion (TAPSE), pulmonary acceleration time/pulmonary ejection time (PAT/PET), and right ventricular fractional area change (RVFAC). The preventive and treatment roles of H_2_ on right heart function were clarified, as described in Fig. 2.

### Inhalation of H_2_ improved the collagen deposition, pulmonary inflammation, and oxidative stress in MMC-induced POVD rat model

**Fig. 3:**
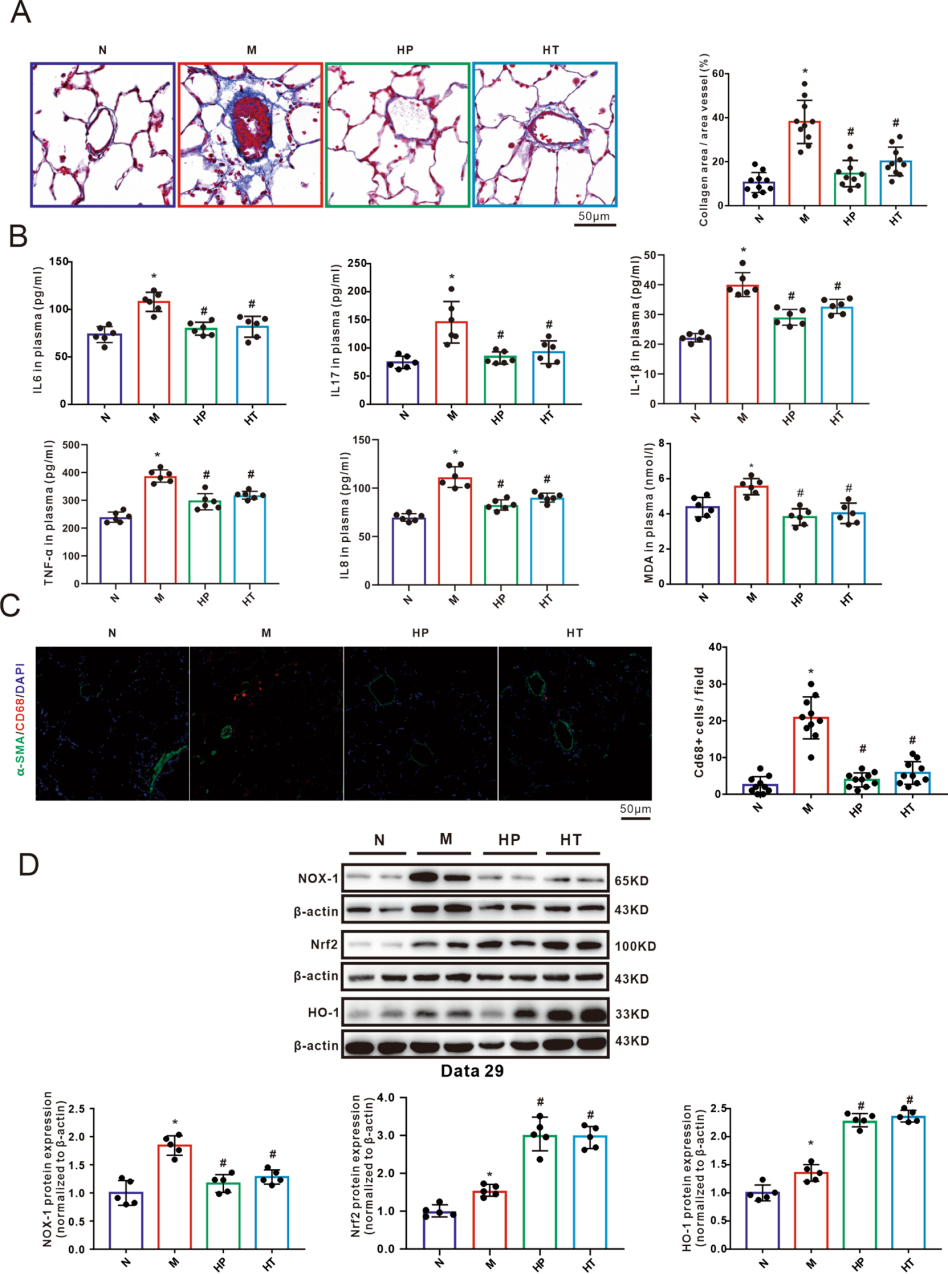
Hydrogen inhalation suppresses inflammation and oxidative stress in PVOD rats. **A**: Representative images of Masson trichrome staining and calculated the collagen area of pulmonary microvessels (*n* = 5 per group, 2 microscopic fields per rat). **B**: The levels of IL-6, IL17 and malondialdehyde (MDA) in plasma (*n* = 6 per group). **C**: Representative images of rat lung sections subjected to immunofluorescence staining with an anti-CD68 antibody(red), anti-α-SMA (green) and DAPI (blue). The CD68 positive macrophages (red) per field were counted from at least nine randomly selected fields under a fluorescence microscope (*n* = 5 per group, 2 microscopic fields per rat). **D**: Western blotting and analysed bar graphs showing the relative protein levels of HO-1, NOX-1. Analysis of western blotting results normalized to β-actin (*n* = 5 per group). All data were presented as means ± SEM, ^*^P < 0.05 vs. Normal, ^#^*P* < 0.05 vs. MMC

Masson’s staining demonstrated that the MMC group exhibited excessive production and deposition of collagen matrix (stained in blue) surrounding the pulmonary vessels compared to the control group. However, inhalation of H_2_ provided protection against these effects (Fig. 3A). As shown in Fig. 3B, the exposure to MMC resulted in significant upregulation of IL-1β, IL6, IL-8, IL17 and TNF-α, indicating MMC-induced systemic inflammation. Moreover, there was an increase in malonaldehyde (MDA), a product of lipid peroxidation, in the MMC-treated rats. However, inhalation of H_2_ demonstrated a remarkable preventive and reversing effect on such increases in inflammatory factors and MDA levels in plasma. This suggests that H_2_ inhalation can effectively inhibit oxidative stress and reduce inflammation in the lungs induced by MMC exposure (Fig. 3B). To further evaluate the role of H_2_ in local pulmonary inflammation, the number of macrophages in each group was assessed by using immunofluorescence staining of CD68 (Fig. 3C). The inducible isoform of heme oxygenase-1, the HO-1, plays critical roles in regulating inflammatory and cytoprotective processes. The enzyme NADPH oxidase 1 (NOX1) is a major producer of superoxide which together with other reactive oxygen species and reactive nitrogen species (ROS/RNS) are major contributors to oxidative damage in pathologic conditions. As shown in the Fig. 3D, after the administration of MMC, there was a substantial increase in the expression of NOX-1, Nrf2 and HO-1. Inhalation of H_2_ gas effectively suppressed the MMC-induced upregulation of NOX-1 in both prevention and treatment group. In both the HP and HT groups, the expression of Nrf2/HO-1 pathway was significantly higher compared to the MMC group.

### H_2_ inhalation reversed the pulmonary vascular remodeling in MMC-induced PVOD rats

**Fig. 4:**
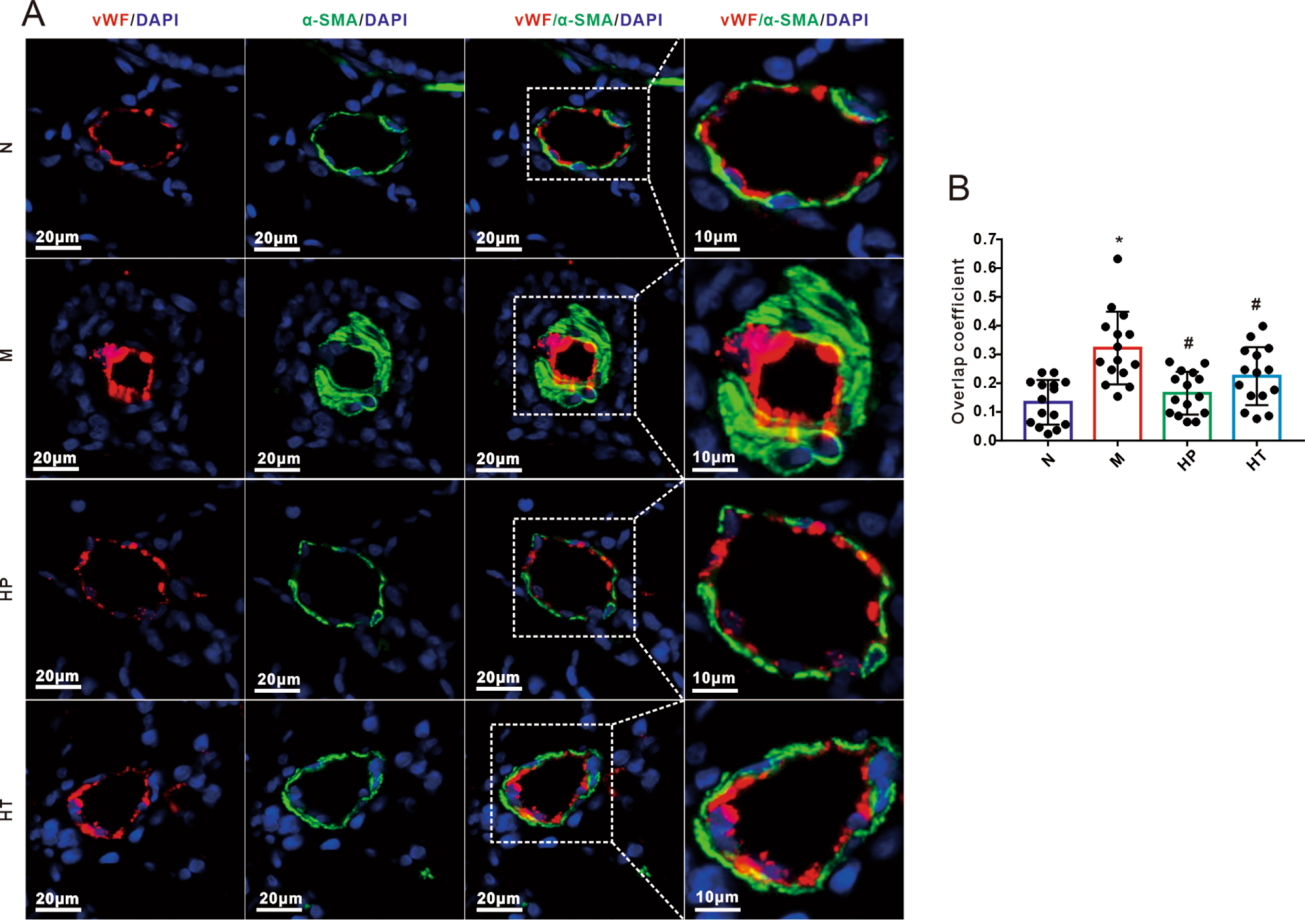
Hydrogen inhalation inhibits the endothelial-to-mesenchymal transition (EndoMT) progression in mitomycin C (MMC)-induced pulmonary veno-occlusive disease (PVOD) rats. **A**: Immunofluorescence staining of lung tissue from rats treated with MMC and/or Hydrogen. The lung sections were stained with vWF (red), α-SMA (green) and DAPI (blue). **B**: Bar graph representing the overlap coefficient of vWF and α-SMA channels reflecting the colocalization rate (*n* = 5 per group, 3 microscopic fields per rat). All data were presented as means ± SEM. Scale bar represented 20 and 10 μm (zoom in) as indicated. One -way ANOVA was used for comparison among four groups. ^*^P < 0.05 vs. Normal, ^#^*P* < 0.05 vs. MMC

To further assess the impact of H_2_ on structural remodeling of pulmonary vessels, we conducted immunofluorescent double-staining using α-SMA (a specific marker for smooth muscle cells, labeled in green) and vWF (von willebrand factor, a specific marker for endothelial cells, labeled in red). As shown in Fig. 4A, compared to the control group, we observed a substantial thickening of the medial wall of pulmonary vascular smooth muscle following exposure to MMC. Additionally, there was a significant overlap between α-SMA and vWF, indicating the presence of endothelial-to-mesenchymal transition (EndoMT). These pathological characteristics collectively represent typical pulmonary vascular changes associated with PVOD. However, inhalation of H_2_ demonstrated a significant restoration of MMC-induced vascular remodeling (Fig. 4B).

### Inhalation of H_2_ preserved MMC-induced EndoMT via rebalance of the Smad signaling pathway

**Fig. 5:**
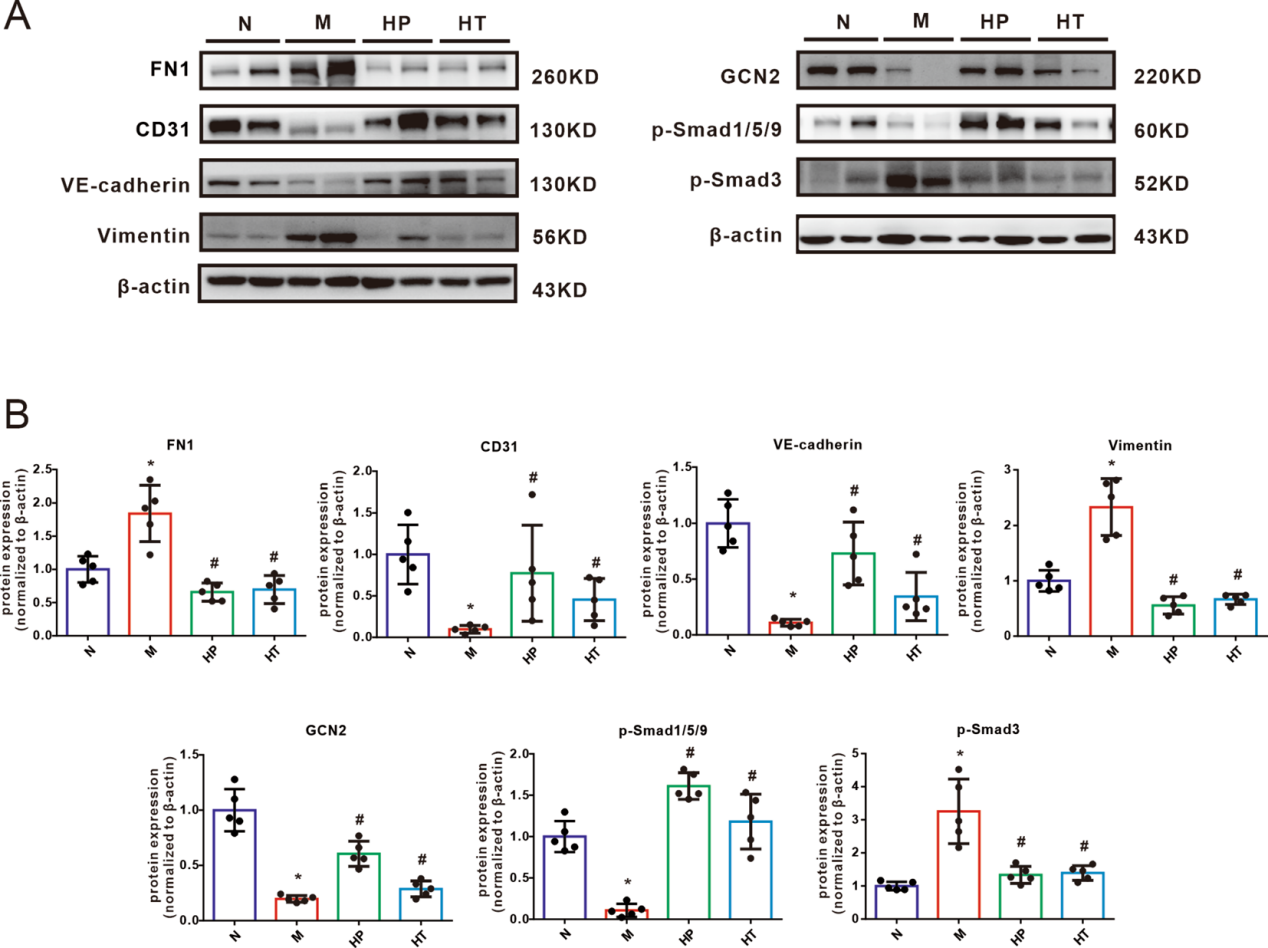
Effects of hydrogen gas on endothelial-to-mesenchymal transition- related proteins in lung tissues from MMC-induced rats exposed to hydrogen. Western blotting (**A**) and analyzed bar graphs (**B**) showing the relative protein levels of FN1, CD31, VE-cadherin, vimentin, p-Smad3, p-Smad1/5/9and GCN2 in the lungs of rats treated with either Normal (N), MMC (M), H_2_ prevention (HP) or H_2_ treatment (HT). Analysis of western blotting results normalized to β-actin. Date was represented as means ± SEM, *n* = 5 in each group. One -way ANOVA was used for comparison among three groups. ^*^P < 0.05 vs. Normal, ^#^*P* < 0.05 vs. MMC

To further investigate the molecular mechanism by which H_2_ prevents and rescues EndoMT in the context of PVOD, the protein expression of classic PVOD-related protein GCN2, mesenchymal markers FN1 and Vimentin, endothelial cell makers CD31 and VE-cadherin, and p-Smads in lung tissue were then measured. The expression of FN1 and Vimentin were increased, and CD31 and VE-cadherin expression were decreased, suggesting obvious EndoMT in PVOD rat model (Fig. 5A and B). These observations support the notion that EndoMT plays a significant role in the development and progression of PVOD. However, we observed a significant reversal in the expression of these EndoMT-related proteins following H_2_ inhalation both in the preventive and treatment way. Furthermore, inhalation of H_2_ effectively inhibited the MMC-induced upregulation of p-Smad1/5/9 and GCN2, while also restoring the MMC-induced reduction of p-Smad3 (Fig. 5A and B). These findings indicate that H_2_ exerts a profound effect on the molecular signaling pathways involved in EndoMT regulation. Specifically, it suppresses the activation of p-Smad1/5/9 and GCN2, while promoting the restoration of p-Smad3 levels. This suggests that H_2_ plays a vital role in preventing and reversing the molecular changes associated with EndoMT in the context of PVOD. Taken together, these data indicated a therapeutic role for H_2_ gas as a prohibitive EndoMT mediator via Smads signaling in MMC-induced PVOD rat model.

## Discussion

In this study, we have provided evidence demonstrating the therapeutic effects of H_2_ on an MMC-induced PVOD rat model. Exposure to a 42% concentration of H_2_ in both the prevention and treatment groups resulted in a significant improvement in survival rates and a reduction in MMC-induced pulmonary vascular lesions. Our findings suggest that Inhalation of H_2_ protects against MMC induced injury through multiple mechanisms. Firstly, it reduces oxidative injury caused by reactive oxygen species (ROS), potentially through its free radical scavenging activities. Secondly, H_2_ protects endothelial cells by suppressing the process of EndoMT induced by MMC. H_2_ also induces the expression of anti-inflammatory and antioxidant factors, including Nrf2/HO-1 and GCN2.

According to the latest clinical diagnosis and treatment guidelines for PH, PVOD is defined as a subtype of PH [1, 14], which is thought to be associated with poor prognosis and low responding to known PH-targeted therapies compared to other forms of PH. Characterized by obliteration of small pulmonary veins by fibrous intimal thickening and patchy capillary proliferation, PVOD may be idiopathic, heritable, or induced by drug/toxin. Recently, numerous evidences have confirmed a causal link between the use of alkylating agents and PVOD pathogenesis [3], such as MMC, a broad-spectrum antitumor agent [15–17]. Inflammatory injury and immune state within pulmonary venules have been considered to play a pivotal role in the pathogenesis of PVOD [18]. The analysis of cytolytic molecular profiling in patients with compartmentalized PVOD revealed substantial alterations in circulating inflammatory cells and the expression of inflammatory cytokines in lung tissue [1, 19].

As a kind of ideal antioxidant, H_2_ exposure has been explored as a potential therapy for various oxidative stress-related diseases with rarely reported side-effects. Preclinical researches and clinical studies have indicated that H_2_ exhibits beneficial effects in mitigating organ damage induced by conditions such as brain and heart ischemia-reperfusion injury, liver injury, diabetes, and COPD [11]. A randomized controlled trial has been conducted by Kang and colleagues, in which, they showed that hydrogen-rich water can decrease the biological reaction to radiation-induced oxidative stress without compromising anti-tumor effects on patients with liver tumors [20]. However, the potential mechanisms leading to these effects remain largely unknown. It has been reported that H_2_ remains non-toxic even at high concentrations [21]. We hypothesized that H_2_ might exert therapeutic effects on MMC-induced PVOD by inducing anti-inflammatory and antioxidant consequences.

H_2_ can diffuse freely into pulmonary tissue and cells with no harm to cellular respiration [22], which acts as a selective antioxidant that specifically neutralizes ·OH and ONOO-, remaining indispensable ROS for physiological signaling [8]. NADPH oxidases are the major source of ROS in vivo in physiological and pathological conditions. NOX-1 is expressed in several tissues including the lung where it seems to be up-regulated in pathological conditions in lung disease. We also found that NOX-1 protein expression was increased in MMC model rats. After inhalation of hydrogen, the expression of NOX1 in lung tissue of rats was significantly decreased.

MDA is a marker commonly used to assess lipid peroxidation and oxidative stress levels. In the rat model of monocrotaline (MCT)-induced pulmonary hypertension, elevated levels of MDA have been reported, indicating increased oxidative stress in this model [23]. In this study, we observed that the levels of circulating MDA were mildly elevated in rats with MMC-induced PVOD. However, after inhalation of H_2_, these levels were significantly restored. This indicates that H_2_ has an anti-oxidative effect, effectively reducing MMC-induced oxidative stress in PVOD. Furthermore, it is well-documented that immune cell infiltration, including macrophages, T lymphocytes, and B lymphocytes, plays a crucial role in the development of pulmonary vascular remodeling in various subtypes of Group 1 pulmonary hypertension [24]. Previous studies have demonstrated that administering hydrogen water to MCT-induced pulmonary hypertension rats can reduce the infiltration of inflammatory cells without causing noticeable side effects [12]. In our study, the number of macrophages was increased and serum levels of IL6 and IL17 were upregulated in the MMC-induced PVOD rats, which were significantly inhibited by prevention or treatment with H_2_ gas inhalation. These results suggested that H_2_ might be an effective therapeutic approach for the treatment of PVOD, through exerting anti-oxidant and anti-inflammatory properties.

HO-1, the inducible isoform of heme oxygenase, is a cytoprotective enzyme that plays a central role in the defense against oxidative and inflammatory insults in the lung. Extensive mechanistic studies have revealed that HO-1 gene regulation responds to positive regulation by Nrf2, which is regarded as a master regulator of the antioxidant response and regulates a series of other genes involved in detoxification [25]. It has been reported that mitochondrial HO-1 fraction is increased in lung tissue when exposed with panoply of stimuli such as hemin, toxicity, oxidative stress [26, 27]. We also found that Nrf2/HO-1 pathway expression was elevated in the MMC-induced rat model (Fig. 3D). MMC-induced HO-1 induction may be cytoprotective against pulmonary inflammation [28, 29]. Inhaled H_2_ induced Nrf2/HO-1 expression and ameliorated lung inflammation and injury in MMC rat models, consistent with other observations [30, 31]. Animal studies have demonstrated that HO-1 plays a critical protective role in several different lung diseases, including pulmonary hypertension, COPD, asthma, and hyperoxic lung injury. Induction of HO-1 may be a beneficial therapeutic strategy against different diseases arising as a result of inappropriate immune response and oxidative dysregulation. Although possible therapeutic approaches to modulate HO-1 expression in patients to combat various oxidative and inflammatory responses include the use of pharmacological agents or gene therapy [28, 32]. Molecular hydrogen, as a non-cytotoxic HO-1 inducer, the use of which might be more easily translated into clinical practice than other therapies.

As is previously reported, PVOD is also affected by heritable genetic mutations. Eyries et al. firstly demonstrated that biallelic mutations of the Eukaryotic Translation Initiation Factor 2 Alpha Kinase 4 (EIF2AK4) gene, which codes for GCN2, is strongly associated with the development of PVOD [33]. Furthermore, the expression of GCN2 was also downregulated in the lung tissue of MMC-induced PVOD rats [5]. The detailed mechanism of GCN2 in the pathogenesis of PVOD remains unclear. The study showed that decreased GCN2 activity may lead to increased susceptibility to oxidation [34] and more intense inflammatory responses by suppressed eIF2a/ATF4/HO-1 signaling pathway [35, 36]. These findings support the involvement of these genes are involved in resistance to oxidative stress and inflammation [37]. Given the anti-inflammatory role of GCN2, the protein expression of GCN2. we observed that H_2_ gas treatment significantly restored the downregulation of GCN2 induced by MMC, suggesting that H_2_ may promote the expression of GCN2. This, in turn, could lead to the activation of the eIF2α/ATF4/HO-1 signaling pathway. These results implied that H_2_ may provide comprehensive protection against vascular remodeling induced by oxidative stress. Ultimately, this could have important implications for the prevention and treatment of PVOD disease progression.

**Fig. 6:**
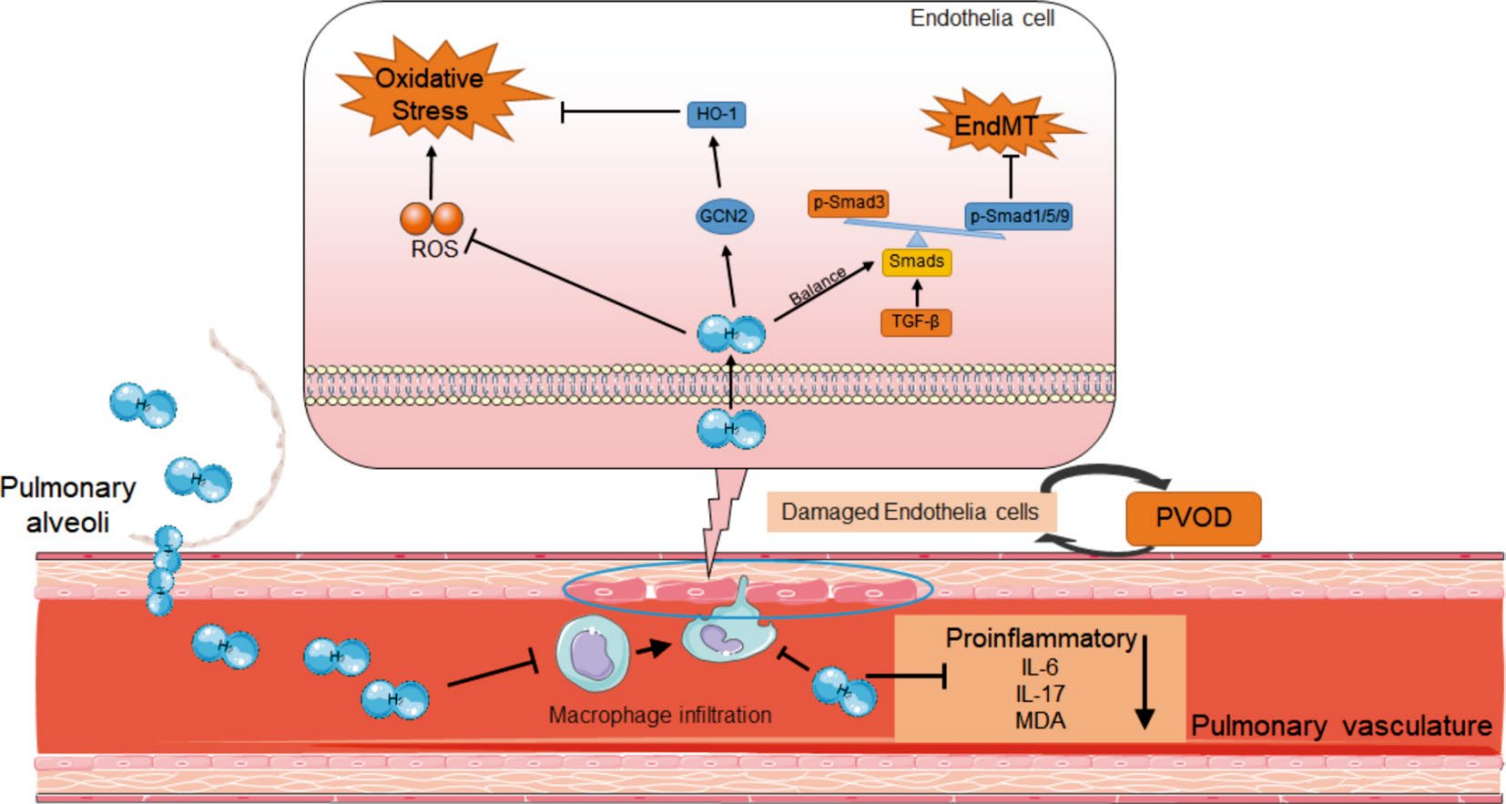
Proposed model of the study illustrating the potential mechanism of molecular hydrogen alleviating PVOD. The inhalation of H_2_ is able to alleviated mitomycin C-induced endothelial-to-mesenchymal transition and oxidative stress, by promoting the expression of the anti-inflammatory GCN-HO pathway, inhibiting the production of inflammatory cytokines in plasma, and balancing the Smads pathway

Recently, EndoMT is deemed to contribute to the pulmonary vascular remodeling during the development of PAH and PVOD [38]. Our previous study has indicated that MMC-induced EndoMT by the upregulation of TGFβ/Smad3/snail signaling axis participate in and contribute to the disease development of PVOD in rats [13]. Moreover, significantly increased phosphorylation of Smad3 and decreased phosphorylation of Smad1/5/9 were detected in MMC-treated rats and PAH patients, suggesting the imbalance of Smad pathways during the PAH process. In the present study, the expression of endothelial cell makers (CD31 and VE-cadherin) and p-Smad1/5/9 in H_2_ inhalation group were significantly increased, compared to solely MMC-treated group. In addition, the mesenchymal markers (FN1 and Vimentin) and p-Smad3 were significantly inhibited after inhalation of H_2_. The EndoMT can be modulated by pathological processes, including inflammation, vascular damage, metabolic dysregulation and so on. Taken together, H_2_ inhalation protected the lung against excessive oxidative stress and inflammation. Additionally, H_2_ inhalation restores the imbalanced Smads signaling pathways, thereby inhibiting EndoMT and preventing vascular remodeling (Fig. 6).

In conclusion, our findings provide evidence that the inhalation of 42% H_2_ has significant benefits in both prevention and treatment protocols for MMC-induced PVOD in a rat model. H_2_ inhalation exerts its therapeutic effects by regulating key factors involved in inflammation, such as GCN2 and HO-1. Furthermore, Inhalation of H_2_ inhibits the process of EndoMT, which is associated with pulmonary vascular remodeling. It is worth noting that MMC, an alkylating agent commonly used in chemotherapy, has been documented to cause lung toxicity and PVOD in patients. Therefore, our study highlights the potential of H_2_ inhalation as a promising approach for mitigating the pathological effects of MMC-induced PVOD and improving patient outcomes [39]. In our study, we propose that prophylactic treatment with inhaled H_2_ has the potential to mitigate the side effects associated with the use of chemotherapeutic agents such as MMC. Additionally, H_2_ inhalation could serve as an adjunctive therapy for patients with PVOD, even in cases where misdiagnosis of PVOD as PH occurs. While further research is needed to fully elucidate the direct effects of H_2_ on PVOD and PH, we believe that Inhalation of H_2_ holds promise as an ideal, effective, and safe therapy that warrants further evaluation. H_2_, as a well-known molecule, may find widespread use in the treatment of PH due to its antioxidant properties and minimal side effects.

### Electronic supplementary material

Below is the link to the electronic supplementary material.

Supplementary Material 1

Supplementary Material 2

## Data Availability

The data that support the findings of this study are available from the corresponding author upon reasonable request.

## Abbreviations

α-SMA: Alpha smooth muscle actin
CO: Cardiac output
COPD: Chronic obstructive pulmonary disease
EndoMT: Endothelial-to-mesenchymal transition HO-1, heme oxygenase-1
MDA: Malonaldehyde
MCT: monocrotaline
MMC: Mitomycin C
NOX1: NADPH oxidase 1
Nrf2: Nuclear factor erythroid 2-related factor-2
PVOD: Pulmonary veno-occlusive disease
PAH: Pulmonary arterial hypertension
PAT: Pulmonary acceleration time
PET: Pulmonary ejection time
RVSP: Right ventricular systolic pressure
RVFAC: Right ventricular fractional area change
ROS: Reactive oxygen species
RNS: Reactive nitrogen species
SPF: Specific pathogen free
TAPSE: Tricuspid annular plane systolic excursion
vWF: Von willebrand factor
