# Supplementary figures and images for: Inhalation of hydrogen gas protects against mitomycin-induced pulmonary veno-occlusive disease
Source: Respir Res. 2024 Jul 16;25:281. doi: 10.1186/s12931-024-02906-y (PMC11253336; doi:10.1186/s12931-024-02906-y)

Figure 3D

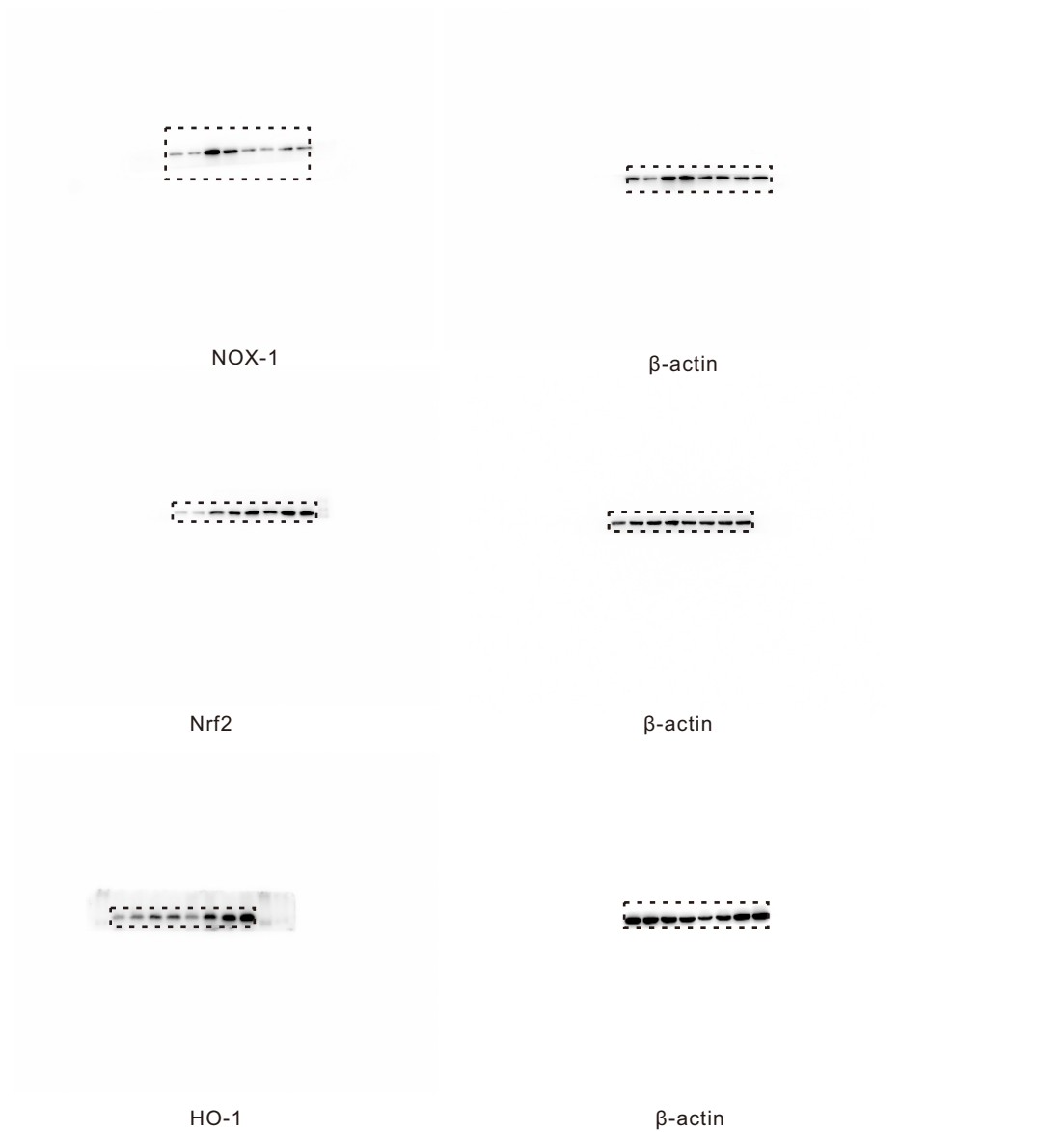

Figure 5A

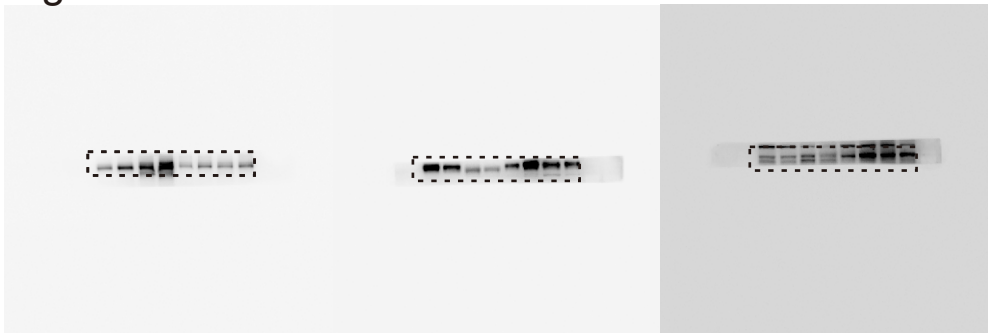

Fn1

CD31

VE-cadherin

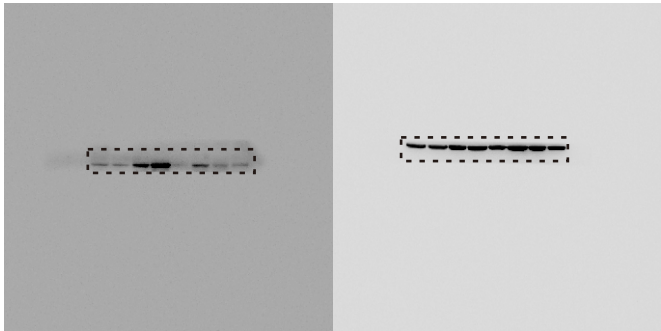

Vimentin

$\beta$ -actin

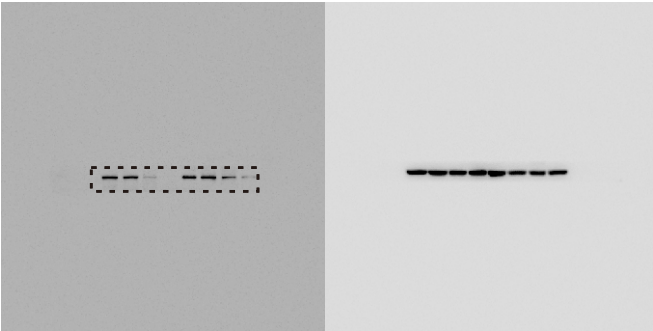

GCN2

$\beta$ -Actin

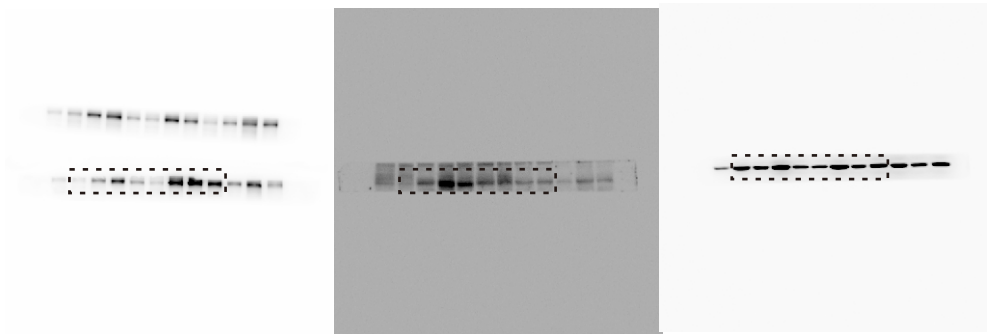

p-Smad1/5/9

p-Smad3

$\beta$ -Actin

Supplement: Supplementary file 1 — Supplementary Material 1 [file 12931_2024_2906_MOESM1_ESM.pdf]
