# Supplementary material for: Inhalation of hydrogen gas protects against mitomycin-induced pulmonary veno-occlusive disease
Source: Respir Res. 2024 Jul 16;25:281. doi: 10.1186/s12931-024-02906-y (PMC11253336; doi:10.1186/s12931-024-02906-y)
Supplement: Supplementary file 2 — Supplementary Material 2 [file 12931_2024_2906_MOESM2_ESM.pdf]

# Figure 1 for response

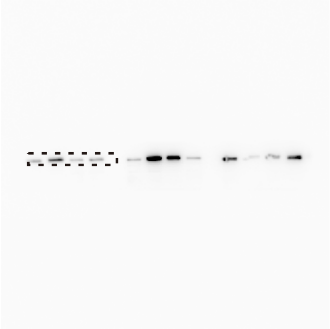

NOX-1

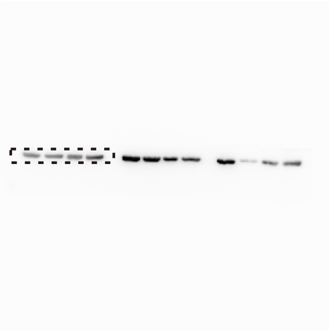

β-actin

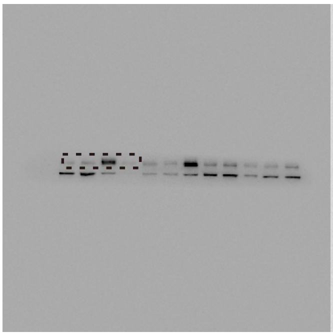

HO-1

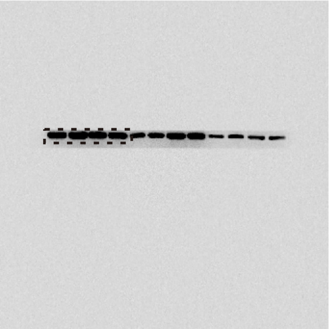

β-actin

# Figure 2 for response

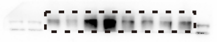

FN1

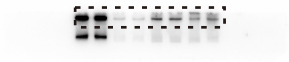

VE-cadherin

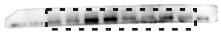

p-Smad3

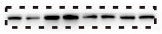

β-actin

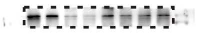

GCN2

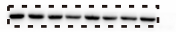

β-actin

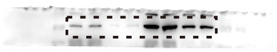

p-Smad1/5/9

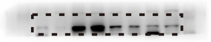

Vimentin

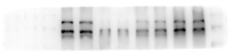

CD31

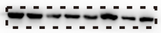

β-actin

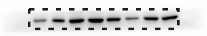

β-actin

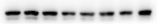

β-actin
